# Three-Dimensional Identification of the Frontal Aslant Tract in the Human Brain: Tractography Study

**DOI:** 10.3390/brainsci16080885

**Published:** 2026-08-20

**Authors:** Sara Kierońska-Siwak, Hanna Mackiewicz-Nartowicz, Anna Sinkiewicz, Agata Kozakiewicz-Rutkowska, Alina Jaroch, Marietta Bracha, Beata Zwierko, Dariusz Grzanka

**Affiliations:** 1 Department of Clinical Pathomorphology, Faculty of Medicine, Collegium Medicum in Bydgoszcz, Nicolaus Copernicus University in Torun, 85-094 Bydgoszcz, Poland; 2 Department of Neurosurgery, Stereotactic and Functional Neurosurgery, University Hospital No 2, 85-168 Bydgoszcz, Poland; 3 Department of Otolaryngology, Phoniatry and Audiology, Collegium Medicum in Bydgoszcz, Nicolaus Copernicus University in Torun, 85-094 Bydgoszcz, Poland; 4 Department of Geriatrics, Faculty of Health Sciences, Collegium Medicum in Bydgoszcz, Nicolaus Copernicus University in Torun, 85-094 Bydgoszcz, Poland; 5 Department of Electroradiology, Faculty of Health Sciences, Collegium Medicum in Bydgoszcz, Nicolaus Copernicus University in Torun, 85-094 Bydgoszcz, Poland

**Keywords:** frontal aslant tract, DTI, probabilistic, deterministic

## Abstract

**Highlights:**

**What are the main findings?**

- The frontal aslant tract was successfully reconstructed in all healthy participants using DTI tractography.
- The potential clinical relevance of probabilistic FAT reconstruction requires validation in neurosurgical populations.

**What are the implications of the main findings?**

- Probabilistic tractography produced more extensive FAT reconstructions than deterministic tractography under the applied protocol.
- Accurate identification of the frontal aslant tract may improve preoperative planning and help preserve language-related white matter pathways during neurosurgical procedures.

**Abstract:**

**Background:** The frontal aslant tract (FAT) is a white matter pathway associated with language and executive functions. The aim of the study was to identify and analyze this tract using DTI tractography. **Methods**: The study included 26 healthy adults. Probabilistic multi-fiber tractography and deterministic single-fiber tractography were applied. The seed region (ROI) was defined in the inferior frontal gyrus, and the target region in the superior frontal cortex. FA, MD, and tract volume were analyzed. **Results**: The FAT was reconstructed in all participants using probabilistic analysis. The left FAT showed higher FA (*p* = 0.012), greater volume (*p* = 0.008), and lower MD (*p* = 0.021) than the right. Probabilistic tractography demonstrated greater tract volume compared to deterministic tractography. **Conclusions**: The FAT can be identified using DTI tractography. Probabilistic tractography produced more extensive FAT reconstructions than deterministic tractography; however, anatomical accuracy was not directly assessed.

## 1. Introduction

The frontal aslant tract (FAT) is a paired white matter pathway connecting the inferior frontal gyrus with medial regions of the frontal lobe, including the supplementary motor area and the medial premotor cortex. The FAT forms part of the frontal connectivity network responsible for integrating language, cognitive, and motor processes [1,2]. This pathway is involved in speech initiation, control of speech fluency, and regulation of executive functions, including response inhibition and action planning. Its dominance in the left hemisphere is particularly significant, reflecting its role in language processing [2,3]. Studies indicate that the FAT plays a key role in coordinating processes related to speech production, such as the initiation of articulation, control of speech rate, and switching between verbal responses. Furthermore, this tract participates in the integration of signals between regions responsible for language processing and structures involved in motor planning and execution. In the right hemisphere, the FAT is primarily associated with executive functions, such as cognitive control and response inhibition [4,5,6].

Damage to the frontal aslant tract may lead to neurological disorders, including postoperative mutism, speech fluency disturbances (e.g., stuttering), and deficits in executive functions. In particular, damage to the left FAT may result in difficulties with speech initiation and impaired fluency. Consequently, numerous neuroimaging studies have examined the correlations between FAT damage and clinical symptoms [7,8].

In recent years, tractography based on diffusion tensor imaging (DTI) has enabled visualization of white matter pathways through the analysis of water molecule diffusion and assessment of nerve fiber integrity. This technique has been applied in studies of the structure and function of the FAT, allowing its three-dimensional reconstruction in vivo. This has made it possible to more precisely define the course of the tract and its correlations with other brain structures [9,10,11].

Previous studies have mainly used single-tensor-based tractography; however, advances in deterministic and probabilistic methods now allow for more accurate reconstruction of complex white matter pathways such as the FAT. Probabilistic tractography has been shown to increase the sensitivity of fiber detection and better represent their course, particularly in regions with complex architecture [12,13].

The aim of the present study was to identify and analyze the frontal aslant tract in healthy adults using diffusion-based tractography. Although leftward FAT asymmetry and methodological differences between deterministic and probabilistic tractography have previously been reported, relatively few studies have directly compared both approaches within the same participants using the same diffusion datasets and anatomically matched ROI definitions. Therefore, the principal contribution of the present study is a controlled within-subject comparison of probabilistic multi-fiber and deterministic single-fiber tractography, together with an assessment of interhemispheric differences in FA, MD, and tract volume [14,15].

## 2. Materials and Methods

### 2.1. Subject

The study included 26 healthy adult participants (aged 34–70 years, 14 women, 12 men) with no history of neurological or psychiatric disorders.

Inclusion criteria were: (1) age ≥ 18 years, (2) no history of neurological disease, (3) absence of cognitive impairment, (4) no speech or language disorders, and (5) normal functioning in executive domains, including response initiation and control.

Exclusion criteria were: (1) age < 18 years, (2) prior diagnosis of neurological or psychiatric disorders, (3) history of brain injury, (4) presence of speech disorders, including stuttering or aphasia, and (5) abnormalities identified on neuroimaging. Characteristics of the study participants are presented in Table 1.

Handedness data were available for all participants. Nineteen participants were right-handed (73.1%), and seven were left-handed (26.9%).

All participants provided informed consent prior to DTI imaging. The study was approved by the local bioethics committee of Collegium Medicum KB316/2022 approved date: 17 May 2022. Imaging data were retrospectively obtained from the database of healthy volunteers at the Department of Otolaryngology, Phoniatry and Audiology at University Hospital No. 2 in Bydgoszcz.

### 2.2. Diffusion Tensor Imaging

MRI examinations were performed using a 3.0 T Philips Ingenia scanner (Philips Healthcare, Philips Medical Systems Nederland) equipped with a 32-channel head coil. The imaging protocol included conventional T1-weighted and T2-weighted anatomical sequences as well as diffusion-weighted imaging (DWI).

Diffusion data were acquired using a single-shot spin-echo echo-planar imaging sequence. The acquisition parameters are summarized in Table 2.

Images were corrected for eddy current-induced distortions and head motion using affine registration. Brain extraction was performed using the Brain Extraction Tool (BET) (https://fsl.fmrib.ox.ac.uk/fsl/docs/structural/bet.html, access on 12 August 2026). Diffusion tensors were estimated voxel-wise to generate fractional anisotropy (FA) and mean diffusivity (MD) maps. The acquired data were used for the reconstruction of white matter fibers, particularly the FAT.

A conventional diffusion tensor imaging framework was selected because the study was based on retrospectively available diffusion data acquired using a standardized clinical MRI protocol. The aim was to compare deterministic and probabilistic reconstruction approaches that could be applied to the same routinely acquired dataset. Advanced diffusion models, such as constrained spherical deconvolution or generalized q-sampling imaging, were therefore beyond the scope of the present study.

### 2.3. Fiber Tractography

Two tractography approaches were used to reconstruct the frontal aslant tract: multi-fiber tractography and single-fiber tractography. In the multi-fiber analysis, the FMRIB Software Library ((FSL, version 6.0.x, Oxford, UK)) was used. DWI data were corrected for head motion and eddy current distortions using affine registration.

All ROIs were manually delineated by the same investigator (S.K.-S.), using predefined anatomical landmarks. The seed ROI was placed in the pars opercularis and pars triangularis of the inferior frontal gyrus, whereas the target ROI included the supplementary motor area and medial superior frontal gyrus. ROIs were defined separately for each hemisphere, and the same anatomical definitions were applied to both tractography approaches. Formal intra-rater and inter-rater reliability analyses were not performed.

The investigator was not formally blinded to participant characteristics; however, ROI placement was performed according to predefined anatomical criteria.

Tractography was performed using a probabilistic diffusion model (BED-POSTX/PROBTRACKX), generating 5000 streamline samples for each seed voxel, with a step length of 0.5 mm and a curvature threshold of 0.2.

In the single-fiber analysis, deterministic tractography based on the diffusion tensor model was performed using DTI-Studio software (version 3.0.3, USA). Tracking parameters included an FA threshold = 0.2 and a maximum angle of 60°.

**Figure 1:**
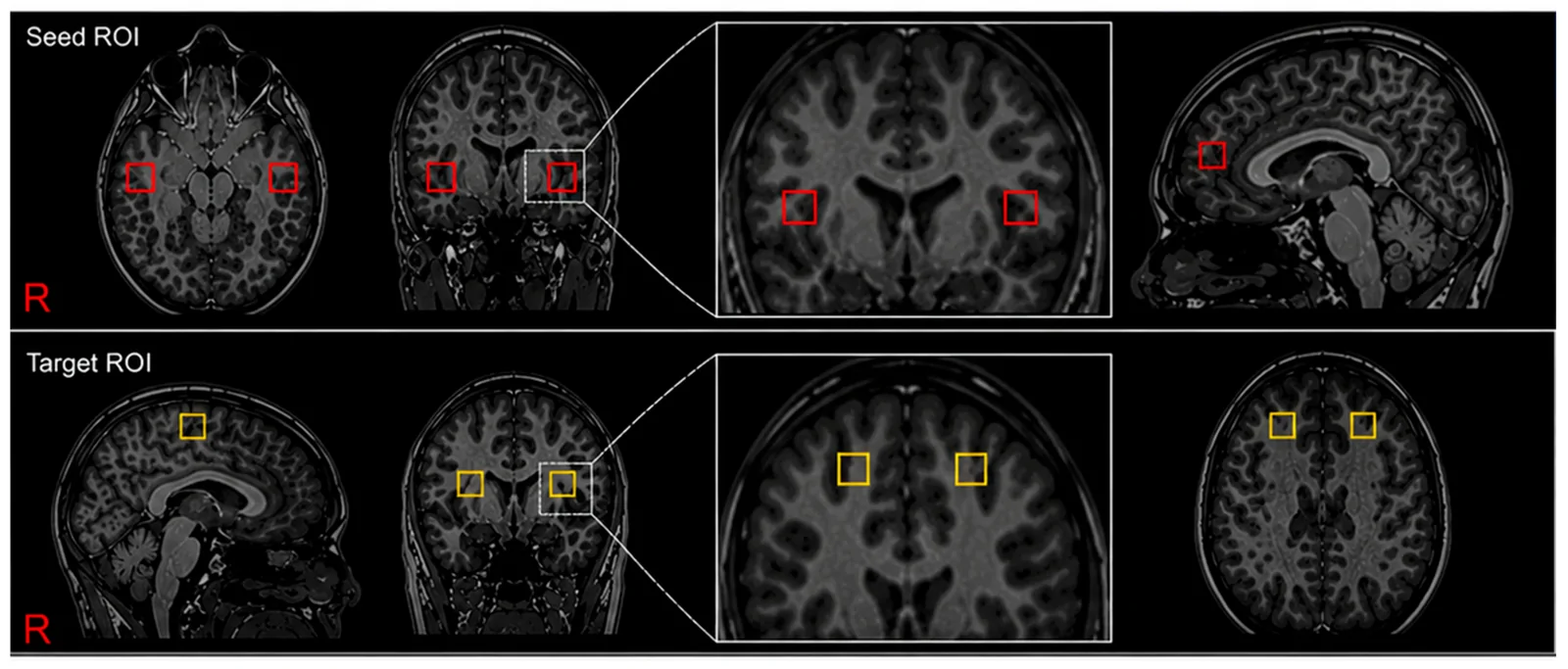
Placement of regions of interest (ROIs) used for frontal aslant tract (FAT) reconstruction. The seed ROI was positioned within the pars opercularis and pars triangularis of the inferior frontal gyrus (IFG), whereas the target ROI was placed in the supplementary motor area (SMA) and medial superior frontal gyrus (SFG). R—right.

ROIs were defined independently for each hemisphere (Figure 1).

### 2.4. Statistical Analysis

Statistical analysis was performed using Statistica 13 software (TIBCO Software Inc., Palo Alto, CA, USA). Data distribution normality was assessed using the Shapiro–Wilk test. Depending on data distribution, paired Student’s *t*-tests or Wilcoxon signed-rank tests were used to compare DTI parameters between hemispheres and tractography methods.

Correlations between age and DTI parameters were evaluated using Pearson’s or Spearman’s correlation coefficients, as appropriate. Additionally, analysis of variance (ANOVA) was performed to assess the effects of sex and age on tract characteristics.

Results were considered statistically significant at *p* < 0.05.

No formal correction for multiple comparisons was applied because the analyses were exploratory. Therefore, the reported *p*-values should be interpreted as unadjusted values, and the findings should be considered hypothesis-generating.

Effect sizes and their confidence intervals were not included in the original statistical analysis. Therefore, the magnitude and precision of the observed differences could not be formally quantified, which should be considered when interpreting the results.

## 3. Results

### 3.1. Probabilistic Tractography

In the present study, the FAT was reconstructed in 26 healthy participants using tractography. The reconstructed FAT originated in the inferior frontal gyrus (IFG) and coursed obliquely upward and medially, terminating in the supplementary motor area (SMA) and the superior frontal gyrus (SFG) in all subjects. All reconstructions demonstrated continuity of fiber trajectories between the defined regions of interest (ROI), and the proportion of successfully reconstructed fibers exceeded 90% relative to the target regions. Mean DTI parameter values for the right FAT were: FA = 0.39 ± 0.05, MD = 1.05 ± 0.14, and tract volume = 185.72 ± 98.34 voxels. Corresponding values for the left FAT were: FA = 0.44 ± 0.06, MD = 0.98 ± 0.12, and tract volume = 232.15 ± 110.27 voxels (Table 3). Statistically significant differences between hemispheres were observed (*p* < 0.05), with the left FAT showing higher FA values, greater tract volume, and lower MD compared to the right FAT. These findings indicate a clear leftward dominance of the tract (Table 4).

### 3.2. Deterministic Tractography

Reconstruction of the FAT using deterministic tractography revealed a similar fiber course to that observed in probabilistic tractography; however, the number of reconstructed fibers and tract volume were lower. The proportion of connections between ROI regions was also lower, ranging from approximately 60–70%. Quantitative measures of FAT reconstruction success are presented in Table 5.

Mean parameter values for the right FAT were: FA = 0.46 ± 0.04, MD = 0.82 ± 0.09, and tract volume = 92.18 ± 41.76 voxels. For the left FAT, the values were: FA = 0.48 ± 0.05, MD = 0.79 ± 0.08, and tract volume = 108.34 ± 52.63 voxels. As in probabilistic tractography, leftward dominance was observed, although interhemispheric differences were less pronounced.

Comparison of methods showed significantly greater tract volume and higher reconstruction efficiency in probabilistic tractography compared to deterministic tractography (*p* < 0.05).

**Figure 2:**
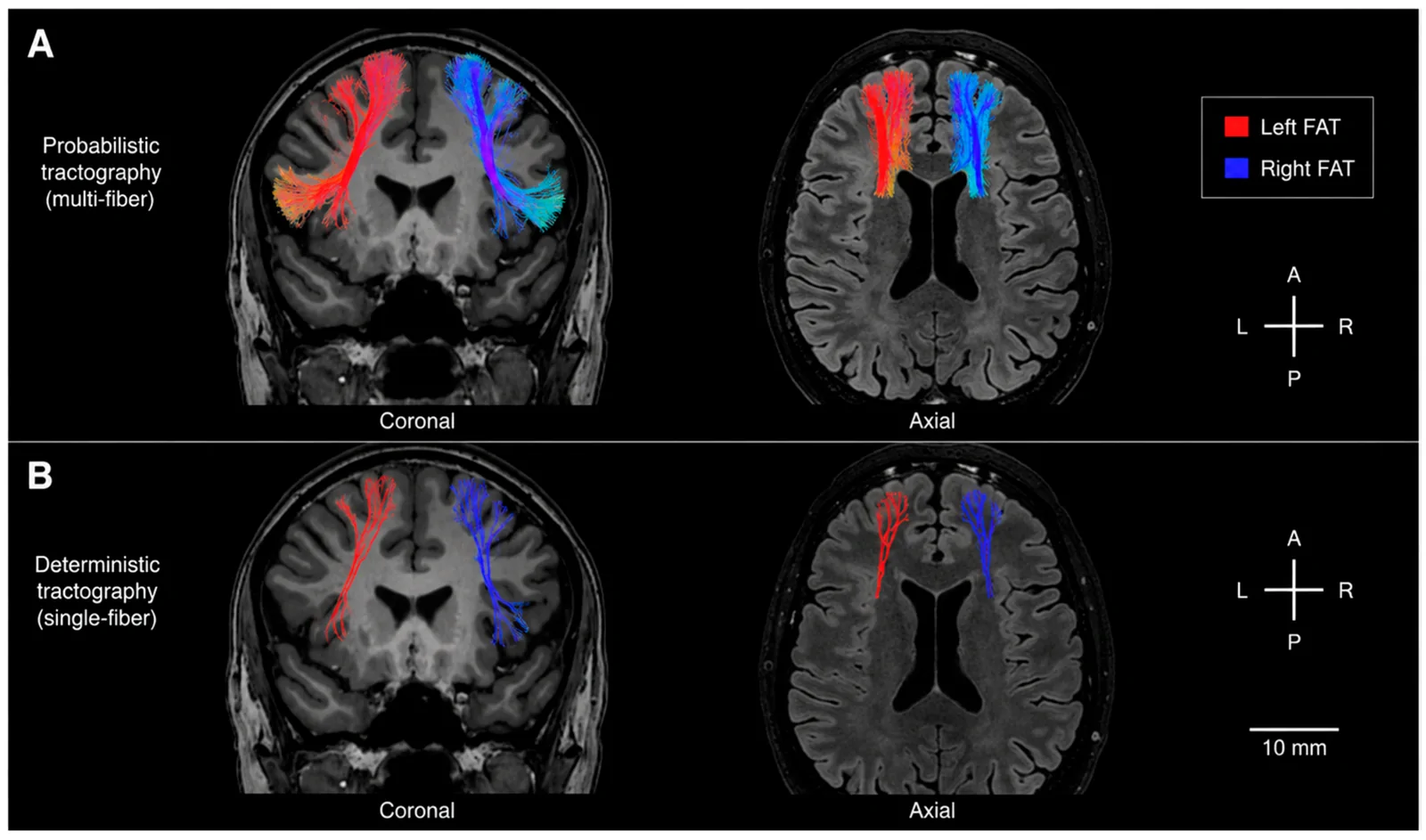
Reconstruction of the frontal aslant tract (FAT) using diffusion tensor imaging tractography. (**A**) Probabilistic multi-fiber tractography. (**B**) Deterministic single-fiber tractography. The left FAT is shown in red and the right FAT in blue. Coronal and axial images are presented in neurological orientation (left = left). A—anterior, P—posterior, L—left, R—right.

Representative reconstructions of the frontal aslant tract obtained using probabilistic and deterministic tractography are presented in Figure 2.

### 3.3. Correlation Analysis

No significant correlation was found between age and FA, MD, or FAT tract volume (*p* > 0.05).

## 4. Discussion

In this study, reconstruction of the FAT was performed in healthy adults using diffusion tensor imaging-based tractography. The results confirm that the FAT can be reliably identified in vivo and demonstrate its characteristic course between the inferior frontal gyrus and medial regions of the frontal lobe, including the supplementary motor area and the superior frontal gyrus [14,16].

The present study confirmed previously reported leftward structural asymmetry of the FAT. The left FAT was characterized by higher FA, greater tract volume, and lower MD than the right FAT. These findings are consistent with previous anatomical studies of hemispheric FAT organization. However, language dominance was not directly assessed in the present study; therefore, the observed structural asymmetry should not be interpreted as direct evidence of functional language lateralization [13,17,18].

The novelty of the present study does not lie in the first demonstration of leftward FAT asymmetry or in the observation that probabilistic tractography produces more extensive reconstructions, as both findings have previously been reported. The principal methodological contribution of this study is the direct within-subject comparison of probabilistic multi-fiber and deterministic single-fiber tractography using the same diffusion datasets, anatomically matched ROI definitions, and a uniform acquisition protocol. This approach minimizes variability related to differences in participant characteristics and imaging acquisition and allows the influence of the reconstruction method itself to be assessed more directly [4,9].

Handedness should also be considered when interpreting hemispheric asymmetry. In the present cohort, 19 participants were right-handed and seven were left-handed. Although leftward FAT asymmetry was observed in the overall study population, language dominance was not directly assessed using functional MRI or another functional method. Therefore, the observed structural asymmetry should not be interpreted as a direct marker of language lateralization. The relatively small number of left-handed participants also precluded robust subgroup comparisons according to handedness [1,19].

The results also confirm that the choice of tractography method significantly affects FAT reconstruction. Probabilistic tractography yielded a greater number of fibers and larger tract volume compared to deterministic tractography. This is consistent with previous reports indicating that probabilistic models better capture the complex architecture of white matter, particularly in regions with crossing fibers. At the same time, deterministic tractography showed higher FA values, which may result from more restrictive reconstruction criteria [7,11,19,20,21].

An important aspect of this study is the lack of a significant correlation between age and FAT DTI parameters within the study group. The literature suggests that significant changes in FA and MD parameters are primarily observed in older populations, indicating the need for further studies including a broader age range [11,22].

The clinical relevance of the FAT has been widely described in the context of speech and executive function disorders. Damage to this pathway may result in postoperative mutism, speech fluency disturbances, and deficits in cognitive control. Therefore, precise identification of the FAT is particularly important in planning neurosurgical procedures, especially within the frontal lobe, where the risk of damaging structures responsible for speech is high [23,24,25].

Despite its important findings, this study has certain limitations. First, the sample size was relatively small, which may limit the generalizability of the results. Second, fiber reconstruction using tractography depends on selected parameters and the definition of regions of interest, which may influence the final representation of the tract. Moreover, although DTI is highly useful, it does not allow for complete representation of all nerve fibers, particularly in regions with complex organization [26].

The observed leftward asymmetry is consistent with previous neuroanatomical and functional studies demonstrating hemispheric specialization of the FAT. Catani et al. reported stronger structural connectivity of the left FAT in relation to language processing, whereas Dick et al. emphasized its role in speech initiation and verbal fluency. Similarly, Landers et al. demonstrated that preservation of the dominant FAT during glioma surgery was associated with better postoperative executive function. Collectively, these observations support the hypothesis that the left FAT undergoes greater functional specialization, which may explain the higher FA values and larger tract volume observed in the present study [2,12,13,27,28].

The results of this study indicate that probabilistic tractography enables more sensitive reconstruction of the FAT compared to deterministic methods. This is due to its ability to model multiple fiber directions within a single voxel, which is particularly important in regions with complex white matter architecture. In contrast, deterministic tractography provides more conservative reconstructions and may underestimate tract volume [29,30].

The present findings have several potential clinical implications. Accurate visualization of the frontal aslant tract is increasingly important during preoperative planning for tumors involving the superior frontal gyrus, supplementary motor area, and inferior frontal gyrus. Preservation of this pathway may reduce the risk of postoperative speech initiation deficits and supplementary motor area syndrome. Furthermore, probabilistic tractography may improve surgical planning in regions containing complex crossing fibers by providing a more comprehensive representation of white matter architecture [31,32].

Beyond preoperative neurosurgical planning, probabilistic tractography may also be useful in research investigating language networks, hemispheric asymmetry, and white matter alterations associated with neurological disorders. Its ability to model multiple fiber orientations within a voxel may be particularly advantageous in regions containing crossing fibers. However, because the present study included only healthy participants and did not involve intraoperative validation, the potential clinical superiority of probabilistic tractography should be confirmed in future studies involving patients with frontal lobe lesions [1,2,3,33].

Although DTI remains the most widely available diffusion technique, it cannot accurately resolve all crossing, kissing, or branching fibers. Consequently, tractography should be interpreted as a computational reconstruction of the most probable fiber pathways rather than a direct anatomical visualization. Advanced diffusion models such as constrained spherical deconvolution or generalized q-sampling imaging may improve reconstruction accuracy in regions of complex white matter architecture [34,35].

Although probabilistic tractography produced larger and more extensive FAT reconstructions than deterministic tractography, this finding should not be interpreted as evidence of greater anatomical accuracy. The present study did not include an anatomical, histological, or intraoperative reference standard. Therefore, the greater tract volume observed with probabilistic tractography may reflect increased sensitivity to possible fiber trajectories, but may also include false-positive streamlines. Conversely, the smaller reconstructions obtained using deterministic tractography may reflect its more conservative tracking criteria rather than inferior anatomical performance [36,37].

In summary, the findings confirm that the frontal aslant tract can be effectively identified using DTI tractography and exhibits clear leftward dominance. These data may serve as a basis for further research on the role of the FAT in language functions and its alterations in neurological disorders and aging processes [14,19,38,39].

## 5. Conclusions

The FAT can be effectively identified in vivo using DTI tractography, demonstrating a characteristic course between the inferior frontal gyrus and medial regions of the frontal lobe. Probabilistic tractography produced larger and more extensive FAT reconstructions than deterministic tractography under the applied protocol. However, in the absence of an independent anatomical or intraoperative reference standard, these findings should not be interpreted as evidence of superior anatomical accuracy. Further validation is required before one method can be considered preferable for clinical applications.

## Figures and Tables

**Table 1:** Baseline characteristics of the study participants.

Characteristic	Value
Participants	26
Age, years, range	34–70
Women (%)	14 (53.8%)
Men (%)	12 (46.2%)
Neurological or psychiatric disorders (%)	0

**Table 2:** Diffusion-weighted imaging acquisition parameters.

Parameter	Value
Scanner and coil	3.0 T Philips Ingenia, 32-channel head coil
Sequence	Single-shot spin-echo EPI
TR/TE	Approximately 3232/85 ms
FOV/matrix	224 × 224 mm/92 × 90
Slice thickness/voxel size	2.5 mm, no gap/2.5 × 2.5 × 2.5 mm^3^
Diffusion scheme	60 directions; b = 0 and 1000 s/mm^2^

**Table 3:** DTI parameters of the frontal aslant tract (FAT) depending on tractography method.

Parameter	Probabilistic (Mean ± SD)	Deterministic (Mean ± SD)	*p*-Value
FA (right hemisphere)	0.39 ± 0.05	0.46 ± 0.04	<0.05
FA (left hemisphere)	0.44 ± 0.06	0.48 ± 0.05	<0.05
MD (right hemisphere)	1.05 ± 0.14	0.82 ± 0.09	<0.05
MD (left hemisphere)	0.98 ± 0.12	0.79 ± 0.08	<0.05
Tract volume (right)	185.72 ± 98.34	92.18 ± 41.76	<0.05
Tract volume (left)	232.15 ± 110.27	108.34 ± 52.63	<0.05

Abbreviations: FA, fractional anisotropy; MD, mean diffusivity.

**Table 4:** Interhemispheric differences in DTI parameters of the FAT.

Parameter	Right Hemisphere (Mean ± SD)	Left Hemisphere (Mean ± SD)	*p*-Value
FA	0.39 ± 0.05	0.44 ± 0.06	0.012
MD	1.05 ± 0.14	0.98 ± 0.12	0.021
Tract volume	185.72 ± 98.34	232.15 ± 110.27	0.008

**Table 5:** FAT reconstruction success.

Method	Connections Reaching the Target ROI
Probabilistic tractography	>90%
Deterministic tractography	Approximately 60–70%

## Data Availability

The data presented in this study are available from the corresponding author upon reasonable request. The data are not publicly available due to privacy and ethical restrictions related to patient confidentiality.
